# The narrow gender gap in hookah smoking behavior among Iranian university students

**DOI:** 10.18332/tpc/191108

**Published:** 2024-07-19

**Authors:** Ramin Shiraly, Aryan Mohamadinezhad

**Affiliations:** 1 Shiraz University of Medical Sciences, Shiraz, Iran

**Keywords:** students, gender gap, waterpipe smoking

**Dear Editor,**

Recreational substance use among undergraduate university students has been a cause of concern globally^1^. Generally, males are much more likely than females to report substance use behaviors. Nevertheless, the changing gender gap in substance use patterns should be monitored and addressed^2,3^. Hookah use has now been widely practiced in different parts of the world, particularly among young adults^4^. There are significant health risks associated with hookah use, including periodontal, pulmonary, cardiovascular, cognitive, and neoplastic diseases^5^.

We conducted a survey on the use of recreational substances being smoked by university students in Shiraz, Iran (Approved by the Ethics Committee of Shiraz University of Medical Sciences). Eligible participants were undergraduate students (including two-year associate degree, Bachelor’s degree, Master’s and doctorate degrees) aged ≥18 years, who were studying in a university during the 2021–2022 academic year. Data were collected via a self-administered questionnaire, which included demographic characteristics and questions about self-reported current (past 30 days), one-year, and lifetime prevalence of use of smoked substances, including cigarettes, hookah, cannabis, opium, heroin, cocaine, and methamphetamine.

Overall, 3610 students from five large universities were recruited through a convenient sampling approach. Enrollment was designed to obtain a representative sample from different educational degrees and disciplinary backgrounds. The mean ± SD age of participants was 23.0 ± 2.7 years. About half (50.6%) were female, 85% were single. Participant education level ranged from associate (31.3%) and Bachelor’s (50%) degrees to Master’s (11.4%) and doctoral degrees (7.3%). The most commonly used substances during the past month, in order of decreasing frequency, were hookah (34.2%), cigarette (12.7%), cannabis (0.6%), and opium (0.5%). Self-reported use of heroin, cocaine, and methamphetamines was negligible (<0.1%). Past-month hookah use was more common among male than female students (42.2% vs 26.3%, p<0.001), single than married students (35.8% vs 23%, p<0.001), and was less common among students of doctoral degree than other degrees (5% vs 36.4%, p<0.001).

One-year prevalence of hookah use among male students (61.2%) was 1.6 times higher than among female students (37.1%, p<0.001), whereas the one-year prevalence of cigarette use among male students (34.9%) was 13 times higher than in female students (2.7%, p<0.001) (Table 1).

**Table 1:** Prevalence and gender ratio of use of recreational smoked substances among university students in Shiraz, Iran (N=3610)

*Cigarette use*	Men* n (%)	Women* n (%)	*Total n (%)*	*M/F ratio (95% CI)*
One month	444 (24.9)	13 (0.7)	457 (12.7)	35.6 (20.1–60.9)
One year	622 (34.9)	49 (2.7)	671 (18.6)	12.9 (9.6–17.5)
Ever	1049 (58.8)	119 (6.5)	1168 (32.3)	9.0 (7.4–11.0)
**Hookah use**				
One month	753 (42.2)	480 (26.3)	1233 (34.2)	1.6 (1.4–1.8)
One year	1091 (61.2)	678 (37.1)	1769 (49.0)	1.6 (1.4–1.8)
Ever	1349 (75.7)	931 (51.0)	2280 (63.2)	1.4 (1.3–1.6)
**Cannabis use**				
One month	17 (0.96)	3 (0.16)	20 (0.6)	5.8 (1.7–19.8)
One year	47 (2.6)	8 (0.4)	55 (1.5)	6.0 (2.8–12.7)
Ever	173 (9.7)	20 (1.1)	193 (5.3)	8.8 (5.5–14.1)
**Opium use**				
One month	16 (0.9)	2 (0.1)	18 (0.5)	8.2 (1.8–35.7)
One year	19 (1.1)	3 (0.2)	22 (0.6)	6.5 (1.9–21.9)
Ever	79 (4.4)	7 (0.4)	86 (2.4)	11.6 (5.3–25.1)

* Gender differences were statistically significant (p<0.001 for all categories).

This study shows a narrow gender gap in hookah use and a large gender gap in the use of cigarettes and illicit smoked substances among Iranian university students. Hookah is usually smoked in groups and may serve as a mode of communication among youth^6^. Furthermore, a popular belief among young adults is that hookah use is a less dangerous and non-addictive method of tobacco smoking^7^. The high prevalence of hookah use among female students^8,9^ is worrying since females tend to progress more rapidly than males from initial use to addiction. Socializing/partying and peer pressure have been reported as the most common motivating factors associated with smoking hookah among university students^10^, which could promote hookah-sharing behaviors and encourage initiating other substances through creating exposure opportunity^11^.

The narrow gender gap in hookah use is a matter of concern because it might be associated with other risky behaviors^12^. It should, therefore, be taken more seriously than just a method of tobacco smoking.

## Data Availability

The datasets generated and analyzed during the current study are available from the corresponding author upon reasonable request.
